# How Do Pharmacists Construct, Facilitate and Consolidate Their Professional Identity?

**DOI:** 10.3390/pharmacy4030023

**Published:** 2016-07-22

**Authors:** Patrick Dawodu, Paul Rutter

**Affiliations:** 1 Faculty of Education, Health and Wellbeing, University of Wolverhampton, Wulfruna St, Wolverhampton WV1 1LY, UK; p.dawodu@wlv.ac.uk; 2 School of Pharmacy and Biomedical Sciences, University of Central Lancashire, Fylde Rd, Preston PR1 2HE, UK

**Keywords:** pharmacy, professional identity, professionalism

## Abstract

The pharmacy profession continues to experience change regarding roles and responsibilities. The supply of medicines still remains a central function, but patient-facing, clinical roles are now becoming more commonplace, where pharmacists use their expert knowledge to maximise patient use of medicines. This transitional state from supplier of medicines to medicines expert raises questions over the contemporary professional identity of pharmacists. This literature-informed commentary highlights the current situation regarding how pharmacists’ identity is formed and reinforced. The authors suggest that the profession needs to be clearer in articulating what pharmacy does, and advocate the need for strong branding that the profession, public and other healthcare practitioners understand.

## 1. Introduction

Technological, social and policy changes within healthcare have altered the role and professional standing of the pharmacist. Notably, the change to mass production of medicines (by the pharmaceutical industry) has eroded the status that was afforded to the profession.

This function of making medicines is now almost redundant and has been replaced predominantly by a supply and sales function, which the majority of pharmacists are actively engaged in performing, and which has drawn criticism as not justifying the pharmacists’ rigorous training [1]. The under-utilisation of pharmacists continues to be recognised by policy makers [2].

To combat this, the profession of pharmacy is trying to champion the pharmacist as the medicine expert, highlighting that the pharmacist is best placed to maximise patient use of medicines and thus contributing to improving patient health and well-being [3,4,5]. The “medicine expert tag” could be said to represent the “pharmacy brand”. In other words, it is the profession’s unique selling point, which no other health care professional can provide.

As many large organisations recognise, having a clear and definite brand is necessary to achieve an impact in terms of sales, profitability and customer loyalty. The brand is jealously guarded and actively promoted, for example, Nike, Coca-Cola and Apple take aggressive stances on brand infringement and run sustained and global marketing campaigns. Parallels can be drawn between commercial branding and the branding of the pharmacy profession; for example, the public, patients and other healthcare workers need to know what pharmacy is, does and can do. We therefore argue that the “pharmacy brand” is vital to the practice of pharmacy, but its “customers” currently poorly understand “the brand”.

Why this is the case is complex and involves a number of inter-related facets; from tension between commercial enterprise and ethical imperative through to societal perception and expectation. However, effective branding is something that can be managed and controlled by those responsible who “sell and maintain” the brand—the profession of pharmacy. This requires the profession to consistently promote this brand, but for this to happen pharmacists need to have a clear sense of who they are and what they stand for. In other words, we are concerned about an aspect of self-identity that has to do with our profession; the need for professional identity.

We argue that there is a lack of consensus to clearly articulate the professional identity of a pharmacist. The literature uses the term “professional” in a variety of contexts [6]. These contexts include professional development, professional socialisation, professional education, professional formation and professional identity [7,8,9]. An ideal profession is said to have its own standards of education, practice is legally recognised by licensure, and the profession is relatively autonomous with members having a strong sense of identity [10]. Using these parameters then pharmacy appears to fulfil many of these in its assertion of qualifying as a profession. Other viewpoints categorising professionalism exist. For example, the structuralist-functionalist perspective, whereby professions are seen as fulfilling certain functions which are vital to the workings of modern industrialised society, and without which these functions would cease to exist [10]. Again, one could argue that pharmacy fulfils this criterion.

Difficulties begin when translating “professionalism” into context specific meaning. In other words, what constitutes “pharmacy professionalism”? To date no consensus exists defining pharmacy professionalism, how this can be engendered in people, or how it might be measured or evaluated. The uncertainty of where pharmacy will sit in healthcare serves to compound these problems. The journey from dispenser of medicines to medicines expert is still evolving, and a road map of how to get to this end point is needed. In our opinion, incoherent, confused and mixed messages conveyed by the profession (about what “*we are*”) are hindering professional socialisation and consequently the ability of “future pharmacists” (students) and qualified pharmacists to gain a sense of true professional identity.

Professional identity has been shown to provide a sense of worth, belonging or purpose [11]. Johnson et al. spoke of “a vocation that we find agreeable to ourselves and may lead to a positive personal and professional image” [12]. A dissonant professional identity has been linked to job frustration, burnout, and attrition [12,13].

The process of becoming a pharmacist and the acquisition of professional identity involves the development of a range of beliefs and attitudes, and an understanding of the boundaries of the role and how to relate to other healthcare professionals as part of a team.

Given the lack of research in medical sociology regarding the professional identity of pharmacists, coupled with current on-going changes within pharmacy, this paper reviews the pharmacy literature with regard to the construction, facilitation and consolidation of professional identity.

## 2. Construction of Pharmacist Identity

At what point does the construction of professional identity begin? Johnson’s notion of “vocation” or calling is exemplified in medicine, where prospective students often have a long-held desire to be a doctor [14]. The embodiment of being a doctor starts early and students align themselves to the principles of what it is to be a doctor. The same cannot be said for pharmacy. At the start of studying pharmacy, many pharmacy students have never worked in a pharmacy environment and for a proportion of students their preferred degree was another healthcare disciple, usually medicine or dentistry.

A body of literature exists looking at reasons and motivating factors as to why students choose to study pharmacy [15,16,17,18,19,20]. For example, Willis et al. surveyed students from 14 UK Schools of Pharmacy and found the strongest motivator for choosing to study pharmacy was that it was a science-based course [17]. Similarly, Keshishian et al. observed pharmacy students were more likely to be interested in science and maths than non-pharmacy students [15]. Capstick et al. found that a large number of students opted first for medicine or dentistry rather than pharmacy, but did report that the most highly ranked motivation was a desire to work in a job where they cared for or helped people [20]. Predictably, the influence of family and friends is an important influence [18]. 

It is clear that “vocation”, or the sense of “wanting to be a pharmacist” is not why students historically or currently choose to study pharmacy. It appears that pharmacy students have an image of the pharmacist—someone who is science-focused, who will make decent money and have a degree of social standing. The concepts of patient care, compassion, empathy and altruism are rarely mentioned. A shift in mind set from the incoming student population is needed to align their perceptions of the pharmacist’s role; if care, compassion and a desire to be the medicine expert is understood early then a sense of belonging and professional identity should be easier to gain.

Currently, it falls on educators to shape student views on what it is to be a pharmacist. Herein lies a major problem. Although curricula across the world are regulated and standards and outcomes stipulated it does not mean that students are taught in the same manner, especially when looking at professionalism and professional identity. In addition, traditional educational programmes may also reinforce the view of a pharmacist as a “scientist” [21,22]. In the US (and to a lesser extent, the UK), attempts have been made to inculcate professionalism and professional identity [23,24,25,26,27]. US Schools of pharmacy have used pharmacist mentors to encourage early adoption of professionalism [24]; Berger describes a School-wide approach to change the ethos of staff and student, from proactive student recruitment and admissions through to exclusion based on behaviours and not just academic performance [25]. Brehm highlighted a multi-disciplinary orientation programme where students from different disciplines had to define elements of professionalism [26]. The use of humanities to exemplify professionalism through classic stories prior to students starting pharmacy school was reported to be well received by students [27]. Just one UK study has reported on how students learn professionalism [23]. This study described professionalism derived via inculcation rather than specific attempts to teach professionalism.

However, it is unclear whether measures taken in US pharmacy schools have had any lasting effect, for example beyond graduation and licensure.

A small body of research does exist that has examined students’ development of identity [28,29,30,31,32]. Generally, findings are not encouraging; Schwirian et al. [28] and Smith [29], both found professional identity to decrease as students progressed through the course, with Shuval [30] noting student expectation (about their future role) lowered as they progressed through the course. Knapp et al. also found that students in the latter part of the course held more negative views toward their chosen occupation than those at the start of the course [31]. Only Hatoum et al. described any positive findings where students had more favourable opinion as they progressed through the course, [32]. Why this has been observed is unclear, although the disparity between educating students for future roles (quite rightly) and the actual day-to-day job they will take on may account for some of the findings. The role of faculty staff, and opportunities to be taught by practicing pharmacists have been reported by students as important in engendering professional identity [23].

## 3. Facilitation and Consolidation of Professional Identity

Evidence suggests that students enter working life without a strong sense of professional identity, and hold negative opinion of the profession. The need to reconcile the professional training of students in pharmacy schools and actual professional practice has been identified [22,23,33]. Early work life and experiences would seem important to enable students to form a clearer sense of professional identity, yet few studies have investigated these formative transition years from graduate to fledgling professional. What studies have been conducted found pharmacists still viewed themselves as primarily “dispensers of medicines”, although pharmacists also saw themselves as medicines advisors, social carer, manager or business person [21,34]. These findings are similar to those of opinion leaders in the profession [35]. Certain factors have been identified that facilitate pharmacists’ identity, and these include practice setting, the skills the pharmacist has acquired, and the influence of mentors [36,37]. Mentorship has shown to be helpful for new pharmacists. Pottie et al. identified that mentorship promoted the mentees image and confidence as well as instilling a sense of worth and motivation [37].

A common theme that seems to run through most studies reviewed is the on-going transition away from the traditional, product-centred role of the pharmacist and the necessity to re-brand as a patient-centred medicines expert. This seems more acute in the community sector where tension exists between retail pharmacy needs and employee pharmacists, and greater role conflict with other healthcare practitioners [38]. The identity of the pharmacist as a medicines expert seems more prominent in hospital practice, as they have a more integrated role alongside other healthcare practitioners [21]. The challenge for the pharmacy profession is to develop a strategy to shift all pharmacists to be seen and act as medicines experts.

## 4. A Possible Future Direction

It is clear that how pharmacists construct, facilitate and consolidate a professional identity is anything but clear. Students, on the whole, have little “calling” to be a pharmacist and, once qualified, find it difficult to develop this identity because of a lack of strong professional leadership and consequently a weak “brand image”. Pharmacy is undoubtedly trying to re-engineer its place in healthcare as the medicines expert but this messaging has a long way to go before being universally recognised. The literature seems to describe a professional identity which is associated with technical skill, but a professional identity which is patient focused is slowly emerging. Completing the transition between these two identities requires a culture shift by most pharmacists—especially community pharmacists where there is a lack of peer support and multi-professional working. Newly qualified and young pharmacists would benefit from appropriate peer/mentor support to develop and facilitate the construction of their professional identity, which more closely aligns to a more patient-facing and clinical role.
